# Applications, Challenges, and Future Directions of Large Language Models in Health Care Communication: Scoping Review

**DOI:** 10.2196/84726

**Published:** 2026-06-26

**Authors:** Jing Chang, Ruotong Peng, Xi Chen, Yishu Zhu, Ruting Miao, Zeng Cao, Hui Feng

**Affiliations:** 1 Xiangya School of Nursing, Central South University, Changsha, China; 2 School of Nursing, Chinese Academy of Medical Sciences & Peking Union Medical College, Beijing, China; 3 School of Nursing, Jiangxi Medical College, Nanchang University, Nanchang, China; 4 Xiangya Hospital, Central South University, , Changsha, China; 5 Xiangya School of Nursing, Central South University, Changsha, Changsha, 410083, China, 1 073182650297

**Keywords:** health care communication, communication accommodation theory, scoping review, digital health, large language models

## Abstract

**Background:**

Effective health care communication is crucial in the medical field. However, effective communication in clinical practice still faces numerous obstacles, and large language models (LLMs) offer various possibilities for improving the quality of medical communication. To date, there are no published reviews on the use of LLMs in health care communication.

**Objective:**

This review sought to summarize the applications and challenges of LLMs in health care communication and to identify directions for future research.

**Methods:**

A comprehensive literature search was conducted in PubMed, Embase, Web of Science, and the Cochrane Library from January 2018 to November 2025. The search and selection process followed the PRISMA-ScR (Preferred Reporting Items for Systematic Reviews and Meta-Analyses extension for Scoping Reviews) guideline and the PRISMA-S (Preferred Reporting Items for Systematic Reviews and Meta-Analyses literature search extension) checklist. Eligible studies used LLMs to facilitate health care communication among the public, patients, and clinicians. Following rigorous data extraction and cross-checking, we conducted a quantitative analysis of characteristics of the included literature. Furthermore, using communication accommodation theory as a framework, we identified application patterns of LLMs in health care communication and summarized current challenges and future directions.

**Results:**

Ninety-six studies were included in this review, all published between 2023 and 2025, summarizing 4 patterns of LLM application in health care communication: transforming medical information (n=30), facilitating dynamic interaction (n=38), empowering communication capabilities (n=10), and optimizing clinical workflows (n=18). The role of LLMs in health care communication is undergoing a paradigm shift from “static information processing” to “dynamic intelligent interaction.” Although they show great promise for practical applications, current evaluation methods and dimensions exhibit significant heterogeneity. Furthermore, LLMs still face multiple challenges in their practical application in health care communication, including technical reliability issues, social trust and adoption, interaction and access barriers, and clinical integration challenges.

**Conclusions:**

Unlike previous studies that merely touched upon the challenges and future directions, this scoping review uses communication accommodation theory to systematically map the application patterns and developmental landscape of LLM-mediated health care communication. Health care communication powered by LLMs holds significant innovation potential and is currently still in the early stages of rapid development. Future research should focus on optimizing model performance, strengthening ethical governance frameworks, enhancing human-machine collaboration models, and ensuring responsible application of LLMs in health care through rigorous empirical validation.

## Introduction

A good clinician-patient relationship is the foundation of medical practice [1]. Effective health care communication not only facilitates interprofessional collaboration and high-quality care delivery but also positively impacts patient treatment adherence, health outcomes, and overall health care quality [2-5]. However, achieving effective communication in clinical practice remains hindered by numerous barriers. Research indicates that clinicians spend more than 20% of their working hours on communication activities, with economic inefficiencies costing approximately US $4.9 billion annually [6]. This significant communication burden and efficiency pressure further limit opportunities for patient-centered interactions [5,6]. Additionally, complex medical terminology and language barriers hinder patients’ comprehension of health care information, negatively impacting treatment decision-making and medication adherence [7,8].

Large language models (LLMs) offer a transformative paradigm for addressing these challenges. As artificial intelligence (AI) systems trained on massive text corpora, LLMs demonstrate exceptional performance in natural language understanding and generation tasks [9,10]. From text analysis and summary generation to clinical applications, LLMs demonstrate diverse capabilities for clinical support [11-13]. Simultaneously, through domain-specific fine-tuning, LLMs can maintain ongoing engagement with user queries, facilitate interactions, and generate controlled outputs [14]. Furthermore, existing research has highlighted the unique value of LLMs in health care communication by developing chatbots with customized behaviors [14]. Multiple studies indicate that LLMs are key tools for improving information transmission efficiency and alleviating the burden of clinical communication [15-18]. Therefore, leveraging LLMs to optimize information dissemination and communication methods will be a crucial approach to improving the quality of health care communication in the future [19].

In this study, health care communication is defined as the dynamic, interactive process within medical settings that facilitates accurate transfer of clinical information, emotional exchange, and collaborative decision-making through linguistic and nonlinguistic mediation [20-22]. To systematically interpret how LLMs intervene in this complex process, this review introduces the interaction-centered communication accommodation theory (CAT) as an analytical framework [23]. Proposed by Howard Giles in 1973, CAT emphasizes that individuals dynamically adjust speech, intonation, and discourse to manage social distance and interpersonal relationships [24,25]. In clinical settings, CAT reveals how providers adapt their communication styles to foster trust and understanding [26,27]. CAT identifies 5 sociolinguistic strategies, including approximation, explicability, interpersonal control, discourse management, and emotional expression [24]. It provides a systematic lens for analyzing how LLMs empower medical communication [28]. Research underscores that CAT uniquely suits large-scale text-based telemedicine among multiple frameworks that elucidate clinical communication [24].

As an innovative and transformative technology, LLM-based health care communication demonstrates immense potential to advance medical communication toward greater efficiency, precision, and personalization [29,30]. However, to date, only one commentary has briefly explored the role and challenges of LLMs in medical communication, highlighting the field’s future potential [31]. At present, the specific use cases, challenges, and future directions of LLMs in current applications remain unclear. Therefore, this study aims to systematically map the current state of research in this field through a scoping review and identify known knowledge gaps. The key issues addressed in this review include (1) systematizing application patterns of LLMs in health care communication based on the CAT, (2) exploring current evaluation methods and dimensions in the health care communication domain, (3) identifying limitations and challenges in applying LLMs to health care communication, and (4) proposing recommendations for future research to inform the better development and application of LLM-based health care communication.

## Methods

### Study Design, Protocol, and Registration

This scoping review adheres to the methodological framework proposed by Arksey and O’Malley [32], and was conducted in accordance with PRISMA-ScR (Preferred Reporting Items for Systematic Reviews and Meta-Analyses extension for Scoping Reviews; Checklist 1) and the PRISMA-S (Preferred Reporting Items for Systematic Reviews and Meta-Analyses literature search extension; Checklist 2) to ensure methodological transparency and reproducibility [33,34]. Our full review protocol is published in the Open Science Framework registries [35].

### Information Sources and Search

To identify relevant English-language literature, the research team systematically searched the PubMed, Embase, Web of Science, and Cochrane Library databases, without using additional information sources. The initial search was conducted on July 30, 2024, and the final search on November 20, 2025, using the same search strategy to identify newly published research. Each database was searched via its web interface, with searches conducted separately within each database. No research registries or other online sources (such as websites, conference proceedings, and journal directories) were searched. We did not contact researchers to seek additional sources. All search strategies were developed specifically for this scoping review and were not formally peer-reviewed by independent experts before implementation. A filter for publications released after 2018 was applied to all databases. The search strategy combined MeSH (Medical Subject Headings) terms with free-text keywords, with core concepts covering LLMs (eg, large language model OR ChatGPT OR large language model* OR language neural network* OR generative AI OR AI-Generated OR generative artificial intelligence OR ChatGPT OR Artificial Intelligence Chatbots OR MedPalm OR GPT OR pretrained model* OR conversational AI OR deep learning language model* OR language model* OR language generation model*) and communication (eg, Respon* OR Repl* OR Report* OR Question* OR Transform* OR Summar* OR Communicat* OR Interpret* OR Explan* OR Inform* OR Answer). The specific search terms were determined by the research team through iterative discussions, with search strategies and keywords adjusted based on results to ensure the retrieval of literature spanning both research domains. We adjusted the search strategy based on each database, and the complete search strategy is detailed in Multimedia Appendix 1 [36-131]. Additionally, we manually screened the reference lists of included studies for additional eligible records. No forward citation searching was performed.

### Selection of Sources of Evidence

The retrieved literature was managed and deduplicated using EndNote 21 software (Clarivate). Two systematically trained researchers independently screened the titles and abstracts of each article based on inclusion and exclusion criteria. Similarly, the full text of articles included in the title and abstract screening was independently reviewed by 2 authors (JC and RP) for inclusion in the evaluation. Any discrepancies were resolved through discussion, involving a third author (HF) when necessary. Additionally, this study aims to provide a comprehensive overview of LLMs’ applications in health care communication. Therefore, the included studies were not assessed for methodological quality to ensure the breadth of the literature review.

### Eligibility Criteria

The review applied the following inclusioncriteria:

1. Peer-reviewed empirical studies applying LLMs to health care communication.
2. Published between 2018 and 2025. This cutoff reflects the introduction of Bidirectional Encoder Representations from Transformers, a novel language representation model widely regarded as the origin of contemporary LLMs [132].

The review applied the following exclusion criteria:

1. Research that focuses solely on LLMs as tools for static knowledge retrieval or question-answering (eg, evaluating only their accuracy in answering questions on a medical licensing exam). This review defines “health care communication” as a collaborative process aimed at achieving information exchange, emotional interaction, and shared decision-making through dynamic interaction. Accuracy assessments based on static question-answering focus solely on the quality of knowledge retrieval; they fail to reflect a model’s ability to respond in real time to user feedback and emotional needs, nor can they evaluate the model’s effectiveness in applying communicative adaptation strategies for dynamic adjustment (for details on excluded categories, refer to Multimedia Appendix 1).
2. Articles unrelated to health care communication, such as prediction or diagnosis of disease.
3. Study protocols, preprints, trial registrations, editorials, letters, and commentaries.

### Data Charting Process and Data Items

The data for each article were extracted through a predesigned data extraction form by the research team. Two authors (JC and RP) independently extracted data from the identified studies using Microsoft Excel, including the author, year, country, study design, medical disciplines, targeted population, research objectives, type of model, evaluation methods, evaluation content, application patterns of LLMs in health care communication, challenges, and other relevant information (eg, barriers). Potential discrepancies in data extraction were discussed by the authors (JC and RP) and resolved. At least one additional author (HF) independently verified the accuracy of each literature record to validate the analysis results.

### Synthesis of Results

This study summarizes and analyzes the extracted data. The research team used descriptive statistical methods to summarize the general characteristics, evaluation methods, and evaluation content of the included studies. Additionally, this study used thematic analysis within the CAT framework to identify patterns and strategies for the application of LLMs in health care communication and to outline the existing challenges. This study strictly followed the 3-stage thematic synthesis method proposed by Thomas and Harden [133]. The 2 authors independently performed open coding of the extracted content from the included studies based on the meaning and content of the data. Subsequently, while remaining faithful to the original findings of the included studies, they distilled their core meanings, categorized the open-coded data, and developed descriptive themes. The descriptive themes were subsequently developed into analytical themes focusing on the patterns and challenges of LLM applications in health care communication. To ensure the rigor of the analysis, all initial coding and the thematic framework were independently reviewed and validated by a third researcher (HF). For any discrepancies in understanding or coding, the research team held multiple meetings for in-depth discussion and, when necessary, redefined the coding manual until the discrepancies were resolved and consensus was reached. The final analysis results were presented through a combination of narrative descriptions and charts, aiming to provide a comprehensive reflection of the current state of this field.

## Results

### Selection of Sources of Evidence

**Figure 1.**
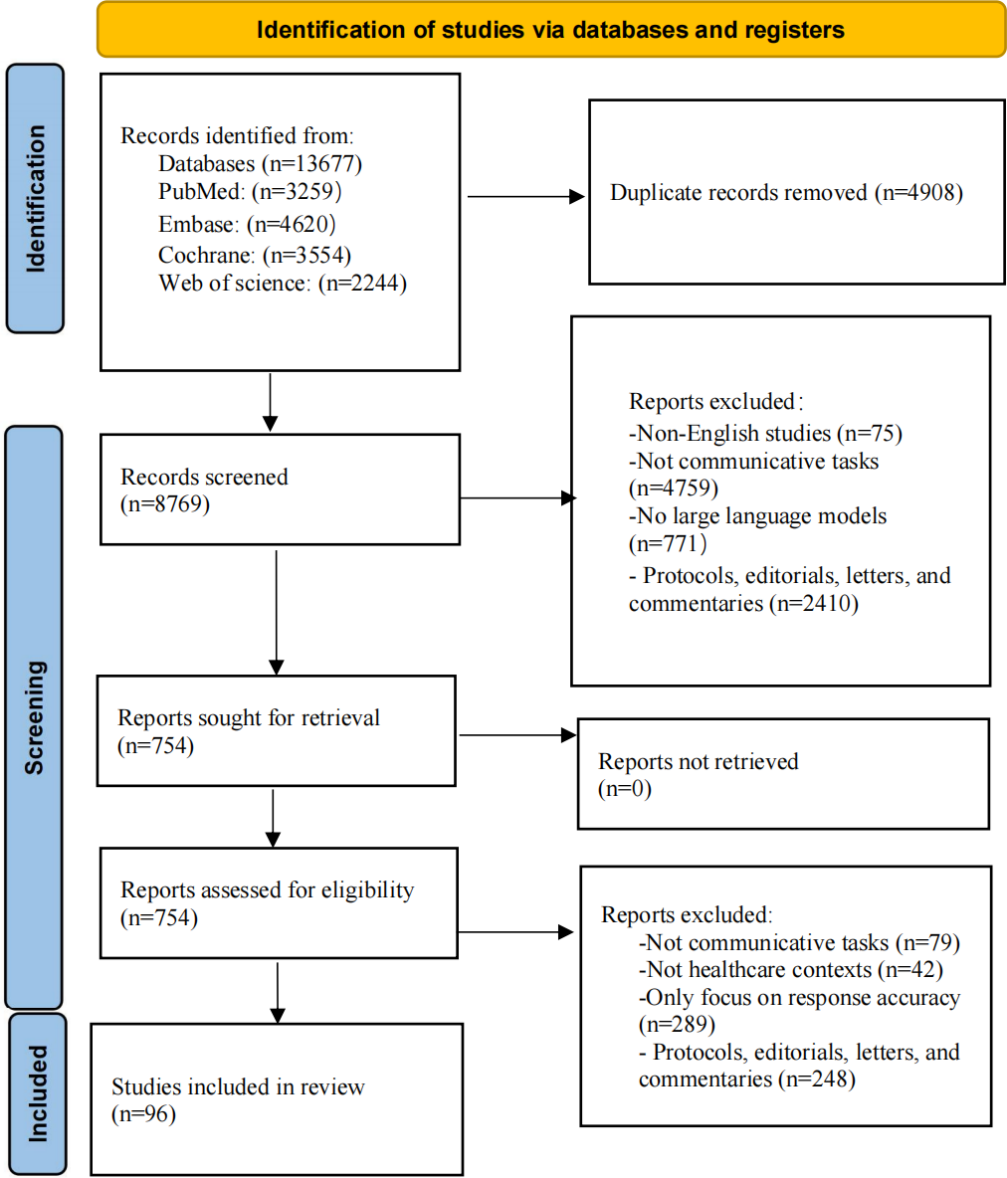
PRISMA (Preferred Reporting Items for Systematic Reviews and Meta-Analyses) flow diagram of article selection.

The study screening process is summarized in the PRISMA (Preferred Reporting Items for Systematic Reviews and Meta-Analyses) flow diagram (Figure 1). A total of 13,677 articles were retrieved from the database, and 4908 duplicates were removed. The full text of 754 articles was screened after screening the titles and abstracts. Finally, a total of 96 studies [36-131] met the inclusion criteria.

### Characteristics of Sources of Evidence

The included studies were published between 2023 and 2025 across 19 countries. The United States had the most studies (n=46) [37-41,44-47,51,53-55,58-60,62,64-66,68,69,73,78,80,83,86,88,94,99,100,102,110,113,116,119,122-131], followed by China (n=13), Turkey (n=10) [70-72,74-76,82,85,89,92], and Germany (n=7) [42,48,50,52,93,103,115]. Other countries included Australia (n=4) [43,101,107,112], South Korea (n=2) [108,117], India (n=2) [49,61], Poland [79], Italy [118], the United Kingdom [36], Ireland [87], Romania [67], the Netherlands [77], Japan [84], Brazil [90], Thailand [120], Sweden [104], Singapore [96], and Colombia [63] (each n=1). The included publications exhibited diverse methodological characteristics, comprising 33 descriptive studies [38,39,41,45,46,49,56,58-60,62,64-66,68,69,71,76,80,82,83,89,94,96,99,100,102,103,115,116,122,126,127], 24 comparative studies [43,47,48,52,61,70,72-75,77,79,81,84,85,87,88,91-93,95,97,98,117], 8 cross-sectional studies [51,54,55,63,78,86,90,125], 6 technology development studies [67,111-113,120,121], 5 randomized controlled trials [37,40,105,114,118], 6 quality improvement studies [44,123,124,128-130], 3 mixed methods studies [108,119,126], and 1 each of proof-of-concept studies [101], observational studies [131], cohort studies[50], qualitative studies [104], exploratory studies [42], pilot studies [36], feasibility studies [109], usability studies [107], multiphase studies [110], multicenter quantitative studies [57], and survey studies [53]. In terms of the specific models used, 83 studies [36-38,40-42,44-53,55-66,68-97,99,100,102-110,112,115-117,119,122,124-131] examined ChatGPT (OpenAI) performance either on its own or in comparison with other models, 6 studies [39,54,98,101,111,123] did not specify the models used, and 4 studies [67,85,113,114] used models they had developed themselves. Research applications spanned multiple medical disciplines, with radiology being the most common field (n=14) [36,37,39-41,45,46,48-50,52-54,65]. For details on the country, study design, medical disciplines, target population, research objectives, model type, evaluation methods, and evaluation content for each study, please refer to Multimedia Appendix 1.

### Synthesis of Results: Different Categories of Application Patterns of LLMs

**Figure 2.**
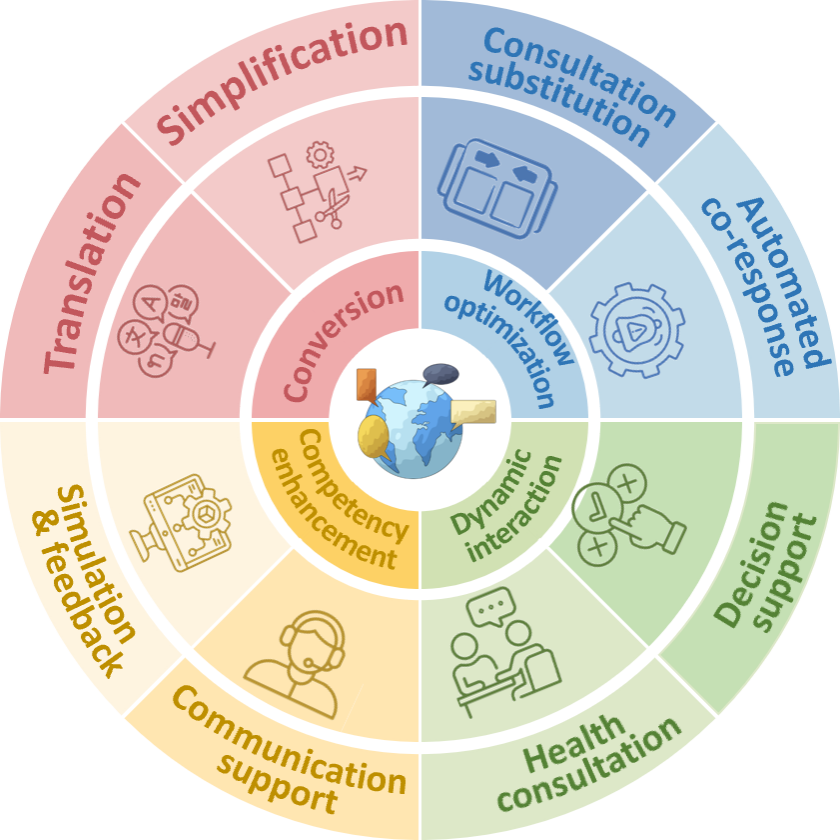
Four application patterns of large language models in health care communication.

The systematic search and screening process identified 96 studies [36-131] meeting the inclusion criteria. Based on the nature of communication tasks, these studies were categorized into four primary application patterns: (1) transforming medical information, (2) facilitating dynamic interaction, (3) empowering communication capabilities, and (4) optimizing clinical workflows (Figure 2). For clarity, Table 1 summarizes the strategies and tactics of CAT, listing their classic definitions and applications in this study.

**Table 1.** CAT^a^ strategies, definitions, and their application [24].

Strategy	Classic CAT definition	Tactics (this study) and application
Approximation	Adjusting one’s communicative style to be more similar (convergence), more distinct (divergence), or unchanged (maintenance) relative to the interlocutor.	Convergence: use language that resonates with users; simplify jargon. Divergence: using technical jargon without a lay explanation. Maintenance: maintain a consistent tone and style regardless of user changes.
Interpretability	Ensuring messages are understandable by adjusting complexity, clarity, or explicitness.	Simplification: breaking down complex medical terms into lay language. Clarification: providing additional explanations or examples to aid understanding.
Discourse management	Regulating conversational flow, including turn-taking and topic control.	Topic initiation: guide the conversation toward relevant topics. Topic shifting: redirect discussions to maintain focus or address new issues.
Interpersonal control	Managing role dynamics and authority in interaction.	Assertiveness: delivering confident recommendations and guiding the consultation. Responsiveness: promptly responding to patient questions and concerns.
Emotional expression	Conveying empathy, support, and emotional alignment.	Empathy: perceiving the patient’s emotions, expressing empathy. Support offer: encouraging the patient, providing emotional support.

a CAT: communication accommodation theory.

**Figure 3.**
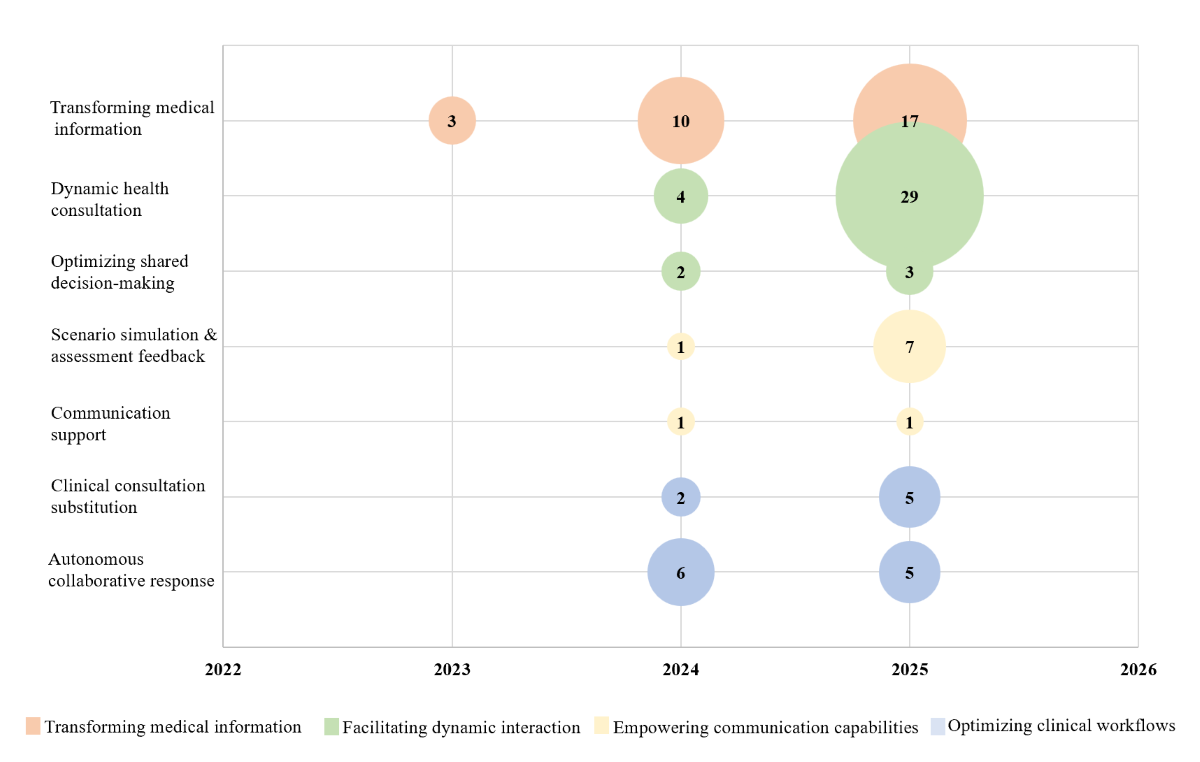
Evolution application patterns of large language models in health care communication. Note: The horizontal axis shows the distribution of publication years, while the vertical axis illustrates the application patterns of large language models in health care communication; the size of the bubbles indicates the number of studies.

The application of LLMs in health care communication has shown a significant growth trend and an evolution in functional focus between 2023 and 2025 (Figure 3). In Figure 3, the horizontal axis represents the year of publication, the vertical axis reflects different application patterns of LLMs in health care communication, and the size of the bubbles indicates the number of related studies. Among these, “Transforming medical information” emerged earliest, with the number of related studies steadily increasing from 3 [41,46,49] in 2023 to 17 [36-40,42-45,47-65] in 2025. The “Facilitating dynamic interaction” domain had no recorded applications in 2023, but rapidly increased to 6 in 2024 and further grew to 32 in 2025, becoming the most widely applied domain to date. Meanwhile, “Optimizing clinical workflow” grew from zero applications in 2023 to 10 in 2025. In contrast, “Empowering communication capabilities” started later (with only 2 studies [107,113] in 2024) but had grown to 8 [104-116] by 2025.

### Transforming Medical Information

LLMs bridge the health literacy gap between physicians and patients by converting specialized medical texts into patient-friendly summaries through interpretability and approximation strategies (n=30). Most studies (n=23) focuses on the “deprofessionalization” of medical documentation. Specifically, LLMs were used to simplify radiology reports (n=13), pathology reports (n=5), and discharge summaries (n=5) [36-58]. Through simplification and clarification tactics, clinical terminology is adapted into plain-language information that patients can readily comprehend, reducing comprehension barriers for those with limited medical knowledge. Furthermore, specialized educational materials (eg, kidney stones, hand surgery, shoulder surgery, and palliative care; n=4) [59-62] and cross-language translations (n=3) were generated [63-65]. This approach mimics the expression patterns of nonspecialist audiences, not only eliminating language and comprehension barriers but also ensuring patients can participate equally in health care decision-making during dynamic clinical interactions, thereby promoting equitable dissemination of health information.

### Facilitating Dynamic Interaction

#### Dynamic Health Consultation

LLMs enable dynamic personalized information exchange through multiturn dialogues, transforming health care information access from “static search” to “dynamic interaction” by leveraging the responsiveness substrategy within interpersonal control strategies (n=33). Thirty-three studies used LLMs to capture patient needs and provide expert-level advice by initiating topics within discourse management strategies, addressing disease-related inquiries [66-98]. For instance, He et al [97] investigated whether LLMs could provide patients with inflammatory bowel disease with appropriate advice comparable to that of gastroenterologists. Wu et al [98] evaluated the quality of preventive and therapeutic advice generated by language models for influenza-related queries in online health communities, focusing on their performance in delivering emotional support through emotional expression strategies.

#### Optimizing Shared Decision-Making

LLMs bridge communication gaps between clinicians and patients by integrating approximation and discourse management strategies (n=5). Two studies used LLMs to provide clinicians with up-to-date clinical guidelines, conversation prompts, and key summaries via approximation strategies, while also helping patients understand disease information, treatment plans, and potential risks to facilitate shared decision-making [99,100]. Three studies examined the use of LLMs to clarify patients’ concerns through interactive dialogue, thereby enhancing the quality of the clinical informed consent process [100-102]. For instance, Allen et al [101] pioneered a 4-phase interactive model (precommunication, language model–guided interaction, clarification phase, and physician review) that uses discourse management strategies to optimize informed consent workflows. Furthermore, this model enables patients to clarify preoperative concerns through repeatable, low-pressure dialogues using support-offer tactics, thereby improving the efficiency of informed consent [101]. The other 2 studies examined LLM support for informed consent in oxytocin-induced labor and total knee arthroplasty [102,103].

### Empowering Communication Capabilities

#### Scenario Simulation and Assessment Feedback

LLMs support patient-centered communication by simulating real-world clinical scenarios, providing constructive feedback, and integrating biosignal technology (n=8). Seven studies used convergence tactics to simulate diverse patient roles, enabling trainees to engage in authentic role-playing across clinical contexts such as pharmacy, emergency medicine, obstetrics and gynecology, nursing education, and pain communication. This approach facilitates mastery of patient-centered communication techniques [104-110]. Some studies integrated virtual reality technology to simulate virtual wards, enabling concurrent training in verbal communication and physical environment management. LLMs further enhance communication capabilities through emotional expression strategies and interpersonal control strategies. Beyond simulation training, LLMs also provide constructive feedback on the quality and emotional resonance of clinical interactions, reinforcing skills such as active listening and empathy. Additionally, advanced systems such as EEG Emotion Copilot integrate biological signals and LLMs to assist clinicians in identifying patients’ emotions, thereby delivering personalized, emotionally intelligent treatment recommendations [111].

#### Communication Support

For individuals with specific language or physical impairments, LLMs provide support by integrating approximation and interpretability strategies, thereby promoting communication equality (n=2). Adikari et al [112] integrated a custom-trained LLM into a conversational system, enabling real-time detection and correction of neologisms and semantic errors, as well as intelligent sentence completion suggestions during interruptions. This effectively leverages strategies to help people with aphasia express themselves in standard contexts, ensuring accurate transmission of meaning. For patients with amyotrophic lateral sclerosis, LLM-driven predictive text functionality significantly reduced the physical burden of eye-tracking operations, while clarification tactics enhanced communication fluency and efficiency in clinical settings [113].

### Optimizing Clinical Workflows

#### Clinical Consultation Substitution

Seven studies explored leveraging discourse management strategies to empower LLMs, either supplementing or replacing health care professionals in specialized applications and triage scenarios, thereby streamlining clinical communication processes (n=7) [113-119]. The “SSPEC” chatbot developed by Wan et al [114] demonstrated its ability to guide patients in recounting medical needs through topic initiation tactics, assist nurses in outpatient reception and triage, and address concerns about model accuracy through response monitoring and early warning mechanisms. In specialized clinical settings, 6 studies integrated interpretability and interpersonal control strategies to enhance the performance of language models in oral hygiene guidance, emergency department management, mental health coping, urology clinical consultations, dental education, and follow-up [115-120]. For example, Chung et al [116] used ChatGPT for preoperative consultations, effectively enhancing patients’ disease awareness and optimizing clinical communication efficiency.

#### Autonomous Collaborative Response

LLMs enhance communication efficiency in health care systems by leveraging interpretability and approximation strategies to automate information generation and enable cross-specialty collaboration (n=11). In prehospital emergency scenarios, LLMs, combined with speech recognition technology, rapidly generate diagnostic summaries to shorten treatment response times [121]. Addressing high-pressure handover environments in emergency departments, Genes et al [122] used LLMs to generate structured transfer summaries, thereby improving emergency department handover processes. Furthermore, this technology enhances interdisciplinary comprehension by adding lay summaries to highly specialized records (eg, ophthalmology notes), ensuring nonspecialist physicians accurately interpret and implement specialist diagnoses during patient referrals [123]. LLMs also demonstrate utility in routine administrative tasks and electronic patient consultations. Specifically, 6 studies explored their capacity for clinical automation through discourse management strategies in automatically responding to patient portal messages and electronic health record inquiries [124-129]. Two additional studies used convergence tactics to ensure generated content aligns with professional norms and expression conventions, examining the “LLM draft-physician review” practice model’s role in reducing health care provider burden and optimizing clinical workflows [130,131].

#### Evaluation Methods and Dimensions of LLM Applications

**Figure 4.**
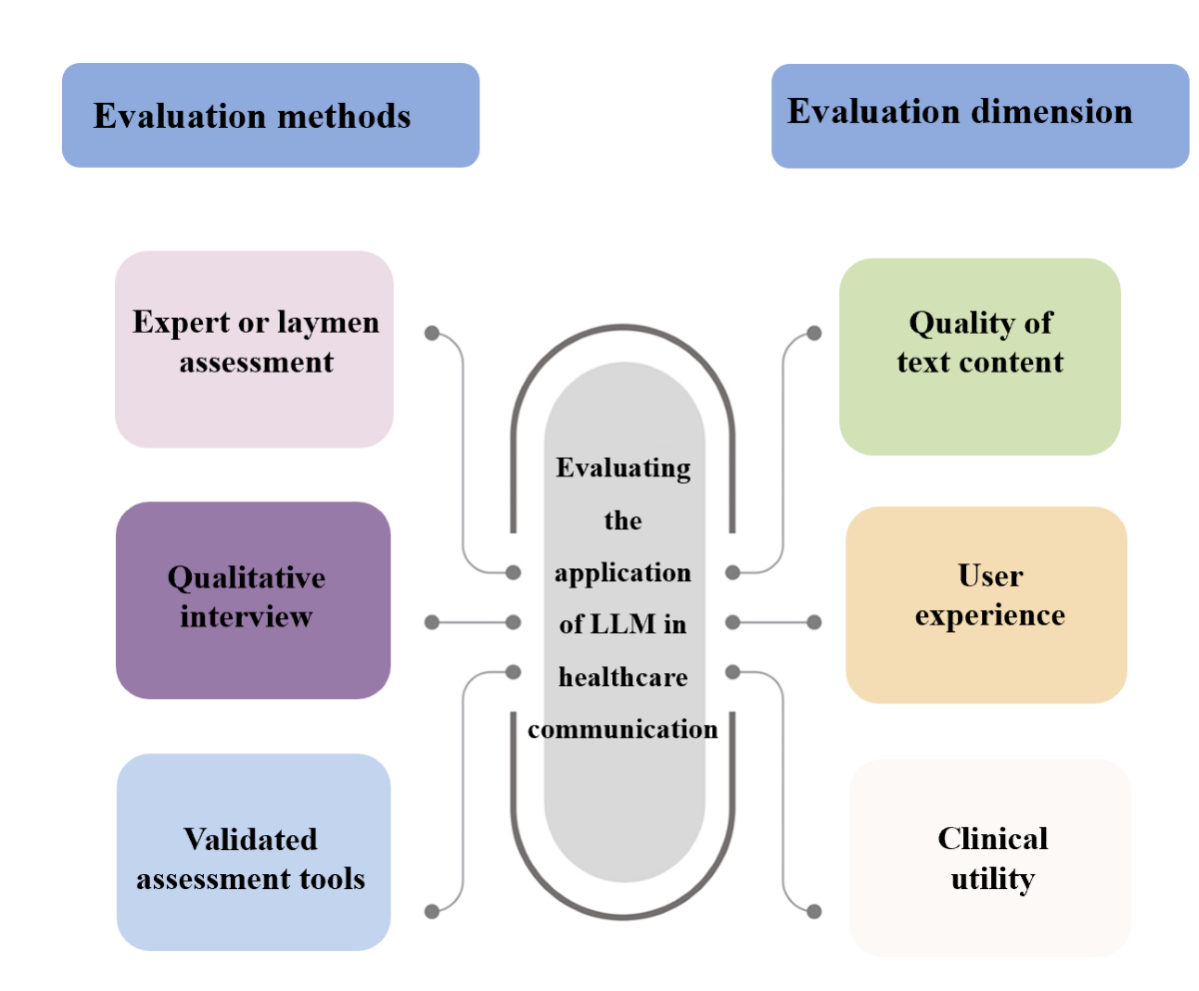
Mapping of evaluation methods and dimensions. LLM: large language model. Note: Validated assessment tools, for example, the Flesch-Kincaid Grade Level is a widely recognized readability formula that estimates the reading level of a text based on average sentence length and vocabulary complexity and generates a corresponding score [77].

Of the 96 studies included [36-131], 93 evaluated the practical applications of LLMs in health care communication [36-66,68-100,102-118,120-131]. Evaluation methods primarily included subjective assessments, qualitative interviews with domain experts and patients, and objective metric measurements using standardized assessment tools (Figure 4).

The evaluation tools exhibited a diverse range of characteristics. Existing studies primarily used Likert scales, custom scoring systems, and questionnaires to evaluate content, combined with qualitative interviews to capture user experience [88,89,110]. A small number of metrics use objective measurement tools; readability metrics include the Flesch Reading Ease, Flesch-Kincaid Grade Level, Coleman-Liau Index, Simplified Nonsense Measure, Gunning-Fog Index, and the Flesch-Szigrist formula [77,85,88]. Some researchers used the DISCERN tool (Deborah Charnock), the Global Quality Score, and the Misinformation Rating Scale to assess content quality [75,90]. Furthermore, measurement tools tailored to specific clinical scenarios, such as the Communication Confidence Self-Assessment Scale and the Mother-Infant Care Communication Assessment Scale, have been used to evaluate changes in users’ communication skills [105,106].

**Figure 5.**
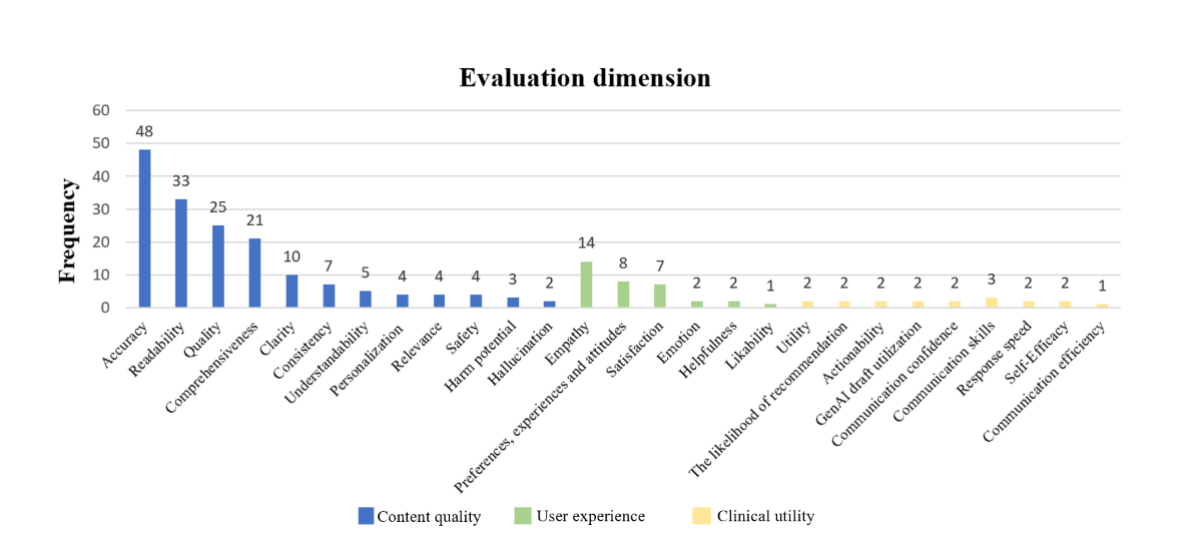
Evaluation dimensions and frequencies. GenAI: generative artificial intelligence.

The evaluation dimensions primarily include content quality, user experience, and clinical utility, with content quality being the core focus of the research (Figure 5). A total of 80 studies used the following criteria to assess content quality: accuracy, consistency, readability, clarity, comprehensiveness, overall quality, safety, and hallucinations [36-66,68-97,99,100,102,103,110,112,114-117,120-123,125-129]. Among these, accuracy (n=48) and readability (n=33) were the most frequently applied evaluation metrics. Twenty-nine studies evaluated users’ experiences with LLM-based health care communication [53-55,61,78,79,81,84,86-88,93,98,104,105,108-110,114,117-119,123,125-130]. These evaluations covered not only metrics such as satisfaction, likability, and practicality but also collected user experiences and subjective preferences through qualitative research [54,55,104,109]. Additionally, some studies assessed model performance from an emotional perspective (such as empathy) [78,79]. Regarding clinical utility, 27 studies examined the feasibility of integrating LLMs into clinical workflows and their impact on medical practice [36,42,47,57,61,75,81,85,87,97,104-109,113-115,117,118,122-124,126,128,131]. Evaluation metrics varied by specific clinical communication task, primarily focusing on feasibility, practical impact, and efficiency. For example, studies used metrics such as willingness to use, usability, usefulness, and applicability to assess feasibility [61,109]. The impact of LLMs as assistive tools on clinical practice was evaluated by assessing improvements in communication confidence, medical history collection, empathy, and communication skills [105,106]. Furthermore, efficiency metrics were primarily measured by quantifying relevant parameters within clinical workflows, including communication efficiency and AI draft usage rates [123,124,129]. For specific details, please refer to Multimedia Appendix 1.

### Comparison of Performance Differences

A total of 19 studies evaluated performance differences among mainstream LLMs in health care communication [48,51,61,63,70,71,73-75,77,85,89-93,95,99,119]. In tasks such as dental implant consultations, melanoma management, patient-physician communication regarding rare diseases, and the interpretation of pathology reports, ChatGPT consistently outperformed Bard (now Gemini; Google) in response accuracy; however, in rhinoplasty consultations, its performance was slightly inferior to Claude’s performance [51,63,73,77,92,93]. In obstetric care consultations, ChatGPT, Kimi (Moonshot AI), and ERNIE Bot (Baidu) showed similar performance in terms of accuracy and completeness [95]. Although ChatGPT demonstrated high accuracy rates in multiple studies, its readability scores were generally low. In contrast, DeepSeek demonstrated advantages in optimizing the accessibility of medical information, with responses that outperformed GPT and Gemini in clarity, comprehensiveness, and readability [70,74,91,92]. In mental health interactions, Pi outperforms ChatGPT in empathy and user acceptance due to its human-like response style [119]. Regarding multilingual performance, ChatGPT performs best at interpreting pathology reports in Spanish, while Perplexity stands out on English tests [119].

Three studies compared the clinical performance of general-purpose models with domain-specific models [84,93,126]. In generating draft responses to patients, ChatGPT significantly outperformed specialized models in overall scores and responsiveness metrics. However, expert evaluations noted that responses from the specialized model CLAIR better aligned with physicians’ professional language styles, whereas ChatGPT’s answers were perceived as having a “robotic” tone [126]. In tasks involving doctor-patient communication regarding rare diseases, ChatGPT outperformed BioMistral. However, BioMistral 7B can run locally within a medical setting, offering privacy advantages [93]. Additionally, Özcivelek and Özcan [85] noted that the domain-specific model Dental GPT demonstrated the highest factual accuracy in consultations regarding oral and maxillofacial prosthetics, but performed worst in terms of readability.

### Existing Challenges

**Figure 6.**
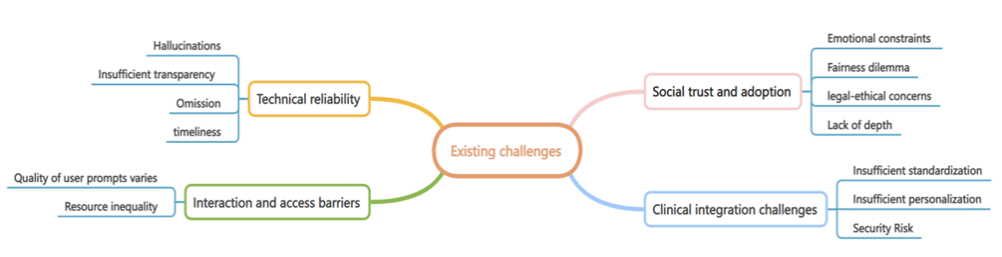
Existing challenges.

We have identified four existing challenges for LLMs in health care communication: (1) technical reliability issues, (2) social trust and adoption, (3) interaction and access barriers, and (4) clinical integration challenges (Figure 6).

### Technical Reliability Issues

Research indicates that, to improve readability for patients, LLMs may oversimplify medical terminology, potentially omitting critical details [58]. Furthermore, constrained by the “hallucination,” models may produce associated inferences with ambiguous terminology definitions, erroneous mechanism descriptions, or insufficient evidence [36,82]. Furthermore, the effectiveness of LLM responses depends heavily on the quality and timeliness of training data. Most current models are trained on general internet corpora rather than rigorously vetted specialized medical datasets, and may generate information that is inconsistent with clinical guidelines or outdated [75]. Combined with inherent “black-box characteristics” and data update delays, this further undermines the interpretability and reliability of their outputs [97,98].

### Social Trust and Adoption

LLMs lack a holistic understanding of patients’ clinical contexts, social backgrounds, and psychological states. Their responses may lack sufficient depth to support the comprehensive situational judgment required in medical practice, making it difficult to address complex clinical issues [87]. At the emotional-interaction level, LLMs cannot perceive nonverbal cues such as facial expressions and tonal shifts, making it difficult to gauge patients’ emotional states and support needs [58]. Moreover, the directive tone and verbose expressions in their responses may provoke resistance among individuals experiencing emotional crises [119]. Furthermore, existing models are predominantly built on English-language datasets and Western value systems, exhibiting significant limitations in multicultural health care settings and potentially leading to biased outputs [128]. Furthermore, unclear legal and ethical accountability, coupled with data privacy and security risks, collectively undermine societal trust in LLMs within serious medical contexts [58].

### Interaction and Access Barriers

The clinical efficacy of LLMs relies heavily on the quality of user prompts [43]. Existing research predominantly uses standardized question sets for testing; yet, real-world clinical inquiries often exhibit ambiguity, emotionality, or unstructured characteristics. Populations with lower health literacy may struggle to obtain accurate information through effective interaction. Additionally, regional disparities in model deployment may create access barriers for economically constrained areas and populations [98].

### Clinical Integration Challenges

Currently, clinical text summaries generated by LLMs often lack consistent formatting and standardization, making them difficult to integrate directly into existing clinical workflows or electronic health record systems [41]. Furthermore, these models tend to provide guideline-based “standardized” responses, struggling to tailor recommendations based on patient history, psychosocial factors, or individual preferences [70,97]. They also fail to dynamically optimize advice based on real-time assessments or rehabilitation progress. More critically, existing models lack mechanisms for medical risk assessment and emergency referral, and are unable to provide timely guidance during patient health crises, posing potential safety risks [119]. The lack of real-world clinical validation further hinders their ability to serve as true clinical substitutes in complex, dynamic doctor-patient interactions [97,121,125].

## Discussion

### Summary of Evidence

This review systematically explores the application patterns of LLMs in health care communication. Existing research has evaluated content quality, clinical utility, and user experience of LLMs primarily through objective and subjective metrics. Despite promising prospects, the field currently faces multiple challenges, which provide direction and focal points for future research. As technology advances and applications expand, LLMs will play an increasingly vital role in health care communication.

### Application Domains and Development Trends of LLMs

Relevant literature was published between 2023 and 2025, coinciding with the November 2022 release of ChatGPT. The geographic distribution of the included studies showed a bias toward developed countries, such as the United States. This may be attributed to the region’s thriving IT industry, advanced health care infrastructure, and ample funding support [134]. ChatGPT emerged as the most frequently cited model in this review due to its practicality and ease of use for clinicians [135]. The studies also encompassed general-purpose models such as Gemini and DeepSeek, alongside a few custom-developed models, highlighting the breadth of models explored in the included research. By testing and comparing different models, researchers can systematically evaluate performance variations and identify model biases [136]. Application domains show diversification trends. As research advances and models optimize, customized professional models focused on clinical specialties are expected to be deployed across multiple scenarios.

The application of LLMs in health care communication is shifting from “static information processing” to “dynamic intelligent interaction.” Transforming medical information is the earliest and most steadily growing area. These tasks rely primarily on structured reasoning and linguistic fluency rather than on complex diagnostic reasoning, giving them an early advantage in technological implementation [137]. As the technology continues to advance, the research focus has shifted toward addressing the more challenging demands of real-time feedback and multiturn dialogue. For instance, the sustained growth in the field of facilitating dynamic interaction indicates that LLMs are gradually evolving into “participants” capable of assisting doctor-patient interactions. Concurrently, the emergence of applications aimed at optimizing clinical workflows reflects a trend toward integrating LLM technology, specifically through the automation of communication tasks to alleviate the increasingly severe administrative burden and clinical pressure within health care systems. Although the field of communication enhancement had a relatively late start, its sustained growth demonstrates that the potential of LLMs to strengthen emotional connections between doctors and patients and assist in complex decision-making is gradually being realized. Future research should focus on translating LLMs’ efficacy into practical clinical settings, particularly on their real-world performance in multicenter, large-scale scenarios [125].

### Main Findings

LLMs empowered by 5 communication accommodation strategies are pioneering new approaches to health care communication. Through strategic language adaptation, they enable dynamic interaction with patients’ needs, demonstrating the potential to bridge gaps in health care resources and improve the quality of care [24]. Current research predominantly focuses on low-level tasks such as text translation and basic consultations, reflecting that LLMs remain in the early stages of integrating into health care communication. Research indicates that simplification and clarification tactics are particularly crucial for addressing the inherent limitations of text-based online consultations [24]. Through these strategies, LLMs simplify medical texts and facilitate cross-language translation, thereby enhancing the transparency of communication between doctors and patients. One study indicates that multimodal LLMs are evolving toward context-aware capabilities [137]. This will help LLMs adjust their communication strategies in real time to achieve “communication adaptation,” further enhancing the interpretability, clinical accuracy, and empathy of the text generated by the models.

The emergence of specialty-specific response chatbots highlights LLMs’ vast potential to improve accessible medical consultations and empower patients’ health management. By incorporating interpersonal control strategies, LLMs are driving a shift in medical interactions from static retrieval to interactive consultation. This strategy is essential for adhering to patient-centered care principles in virtual consultations [138]. In stigmatized domains such as sexually transmitted diseases and mental disorders, LLMs leverage anonymity and accessibility to create low-pressure communication environments [139]. This effectively reduces patients’ need for impression management and fear of self-disclosure, encouraging authentic and in-depth expression [140]. As communication intermediaries, LLMs optimize the understanding and sharing of information through approximation strategies, thereby supporting shared decision-making between doctors and patients [103]. Research has shown that when communication methods align with patient needs, the effectiveness of consultations is significantly enhanced [138]. Future research should quantify the substantive impact of LLM interventions on decision conflict and clinician-patient trust. Given insufficient clinical validation, LLMs cannot replace clinical judgment. Still, they should serve as “communication copilots,” improving medical communication through collaborative methods such as dynamic dialogue monitoring and refining medical history details [103]. Subsequent studies can focus on developing clinically adapted interactive tools to achieve optimal human-machine collaboration [141].

High-fidelity clinical scenario simulations generated by LLMs offer a low-cost, highly scalable solution for communication skills training. By dynamically simulating diverse patient profiles, LLMs adapt their communication to align with users’ language and needs, fostering deeper understanding and connection [24]. However, existing systems are still in their early stages of development, exhibiting limitations in processing nonverbal cues and in addressing privacy and security risks [142]. Consequently, virtual training should be conducted under human supervision, with continuous iteration based on professional feedback to achieve rapid improvement. Future efforts must focus on enhancing the reliability, safety, and scientific validity of virtual patients [143]. Furthermore, relying solely on automated assessment for communication skills may overlook students’ psychological and emotional needs [143]. Therefore, establishing a “human-machine collaborative” assessment system is essential to balance teaching efficiency with humanistic care [142,144]. Additionally, the development of customized intervention programs for individuals with language or motor impairments that incorporate interpretability strategies holds profound significance for enhancing the communication autonomy of this marginalized patient population, thereby improving their social participation, vocational integration, and quality of life [112,113]. It is crucial to emphasize that patient-facing tools must be designed within reasonable parameters and built with appropriate safeguards to ensure safety [54].

LLMs demonstrate application value in optimizing clinical workflows, with their core lying in deep integration with clinical dialogue. Preconsultation effectively enhances patient disease awareness [145]. LLMs empowered by discourse management strategies can serve as foundational consulting tools to alleviate time pressures in clinical work and enhance the efficiency of in-person consultations [24]. Research has shown that effective discourse management strategies can maintain conversational coherence and address patient concerns, thereby fostering trust between physicians and patients in digital settings [138]. However, the absence of nonverbal cues can hinder effective communication, necessitating greater linguistic flexibility and highlighting the importance of integrating convergence tactics into communication systems [24]. Interdisciplinary summaries and automated responses generated through such strategies have demonstratedtheir potential to enhance collaborative efficiency. Although LLMs show potential in simulated treatment dialogues, there is currently a lack of empirical evaluation of their effectiveness in real clinical settings [146]. Therefore, LLMs should currently be positioned as clinical adjunct tools. Their successful deployment may be constrained by existing clinical workflows and health care systems’ capacity to integrate novel communication tools alongside training resources [147,148]. To fully unlock the medical benefits of LLMs, forward-looking policy frameworks and industry standards must be established to ensure their effective integration with clinical practice.

### Evaluation Methods and Dimensions

There is significant heterogeneity in the evaluation of health care communication research using LLMs, making it difficult to accurately assess the models’ task performance and practical effectiveness and obscuring their potential in clinical practice.

Currently, expert evaluation remains the primary assessment method in this field, with only a few metrics evaluated using validated tools. Most studies focus on subjective metrics and user experience, lacking objective quantitative metrics and evaluation tools, which limits the comparability of research findings. In response, some scholars have proposed advancing the quantification and structuring of evaluation methods. For example, Huo et al [149] suggested developing and applying quantitative metrics to evaluate model outputs. Furthermore, the lack of a unified, standardized evaluation framework has led to significant heterogeneity in existing evaluation tools and dimensions, further hindering effective comparisons across studies [150]. Wei et al [151] provided insights for establishing evaluation guidelines for LLMs in medical response by integrating factors such as model versions and prompt design. Future work should prioritize developing standardized evaluation frameworks tailored to medical contexts and exploring hybrid assessment methods that combine human expert reviews, user feedback, and automated metrics.

Regarding evaluation content, existing research primarily focuses on content quality, clinical utility, and user experience [146]. However, metrics concerning ethical considerations, such as fairness and bias, remain underevaluated. Bedi et al [146] emphasize that incorporating bias into evaluation frameworks can be an effective way to mitigate harmful biases in LLMs. Traditional model evaluation has primarily focused on the accuracy of medical question-answering tasks; however, due to the lack of objective metrics, it is difficult to comprehensively assess the true effectiveness of LLMs in complex medical communication scenarios [68,152]. Overall, current evaluation practices for LLM-based health care communication lack rigor. More controlled methods are needed to enhance scalability and scientific rigor, such as using validated tools, clearly defining evaluation dimensions, standardizing assessment criteria, and systematically examining changes in patient behavior or clinical outcomes [143]. Therefore, future evaluation systems must balance technical performance, clinical effectiveness, and ethical compliance to establish comprehensive and reliable metrics for the responsible application of LLMs in health care.

### Comparison of Performance Differences

Research indicates that performance differences among models stem from heterogeneity in their training datasets, algorithmic architectures, and underlying model capabilities [16,153]. Consistent with previous studies, ChatGPT outperforms most general-purpose models in response accuracy, but its limited readability limits its practical application [154]. However, the success of medical communication depends not only on the accurate transmission of information but also on whether that information can be translated into advice that patients can understand and act upon [155].

Across different clinical specialties, model performance exhibits heterogeneity. Compared to domain-specific fine-tuned models, general-purpose models may exhibit reduced reliability in highly specialized contexts due to a lack of specific training [156]. Taking Dental GPT as an example, this model demonstrates high accuracy and relatively low readability in the field of oral and maxillofacial prosthetics [85]. This aligns with previous research indicating that chatbots trained on domain-specific datasets outperform general-purpose language models [157]. This may be because the model was developed specifically for the dental field; the highly specialized training dataset ensures the model’s precise grasp of complex medical facts, though it may also introduce comprehension barriers due to specialized terminology. The CLAIR series of models used by Liu et al [126] demonstrated greater empathy in generating draft patient responses, attributed to the model’s fine-tuning on real clinical scenario data. This helps the model mimic clinicians’ communication styles and care practices in actual practice, achieving a higher degree of clinical realism [126].

Additionally, linguistic differences can also impact model performance. Recent studies indicate that factors influencing LLM performance include not only model capacity but also linguistic diversity, contextual nuances, and the interaction of multimodal content [158,159]. The diversity of clinical specialties and languages underscores the urgent need to develop specialized, multilingual models [160]. Future research should not be limited to increasing model capacity but should also focus on balancing medical authority with public readability through domain-specific fine-tuning, thereby bridging the gap between professional depth and human communication [161].

### Challenges and Future Directions

Overall, we identified existing challenges and recommended ways to address them in the future. A summary of these recommendations is presented in Multimedia Appendix 1.

Concerns regarding the clinical reliability of models arise from issues such as “hallucinations,” “black-box nature,” and information omissions. Research indicates that the quality of model responses is highly dependent on the training data [162]. Therefore, integrating knowledge graphs with multimodal data to construct standardized training corpora holds promise as an effective solution. Knowledge graphs ensure the accuracy and security of data sources [163]. Thefusion of multimodal data facilitates comprehensive analysis of patient information, enhancing LLMs’ capabilities in complex clinical consultations [164]. Furthermore, given the dynamic evolution of medical knowledge, incorporating retrieval-augmented generation frameworks not only dynamically integrates the latest clinical guidelines and medical evidence to improve the timeliness of model outputs but also enhances system transparency [165].

Limitations in emotional interaction and insufficient depth of expression, as well as legal-ethical concerns, represent significant bottlenecks affecting the social adoption and trust in LLMs. Future research may explore multimodal technologies integrating speech recognition, facial expression analysis, and text comprehension with “human-machine collaboration” models to build context-adaptive, empathetic interaction systems [166]. The HAILEY system, developed by Sharma et al [167], provides empathy-based communication suggestions for physicians, precisely supporting medical interactions. Simultaneously, there is an urgent need to build culturally adapted regional corpora to enhance the representation of marginalized groups and eliminate systemic biases [168]. Additionally, multiple studies emphasize the importance of establishing a comprehensive legal framework [169]. Regarding data governance, strict safeguards for patients’ rights to informed consent, data access, and data deletion must be implemented alongside rigorous access control and verification mechanisms [170]. Differential privacy technology, which ensures that individual data remains unidentifiable by introducing controlled noise, represents an effective solution [171].

To overcome interaction and access barriers, future research should focus on developing structured question-assistance frameworks and prompt optimization to guide users in providing structured, complete information, thereby reducing cognitive load during model interactions [172]. Digital inclusion remains paramount, as significant racial, gender, and educational disparities persist in internet access and digital literacy [173]. We therefore advocate for developing training programs to enhance clinicians’ human-machine collaboration skills and implementing public digital literacy education to advance health equity.

LLMs face multiple limitations in clinical interactions. Model outputs should adhere to standardized templates and structured guidelines to ensure consistency and completeness [174]. With explicit authorization and strict privacy safeguards, granting secure model access to patient electronic health records may be a strategy for personalization [175]. Additionally, real-time, multitiered risk identification and response mechanisms should be established, integrating multimodal interaction capabilities to detect emotional shifts or psychological issues [119]. Ultimately, establishing channels for human intervention in scenarios involving complex decision-making or deep emotional support will systematically enhance the applicability and safety of models in clinical settings [166].

In summary, unlike previous studies that merely touched upon the challenges and future directions, this study marks the first systematic application of CAT to research on the use of LLMs in health care communication. It provides a comprehensive overview of the current state of applications and prospects in this field. The study lays the groundwork to unlock the potential of LLMs to optimize communication and to promote their responsible use and high-quality development in clinical practice.

### Limitations

Several limitations of the scoping review must be acknowledged. First, given the inherent conceptual breadth and interdisciplinary nature of LLMs, coupled with the rapid evolution of related concepts, it remains challenging to completely rule out the possibility of omissions despite the comprehensive retrieval strategy used in this study. This study did not use quantitative measures such as Cohen kappa to assess coding consistency; therefore, it has limitations in reflecting statistical reliability among coders [176]. Additionally, excluding non-English literature may introduce selection bias. Second, most studies originate from high-income countries, such as the United States. This not only influences the scope of ethical discussions and the methods used to address specific issues but also raises questions about the applicability of these findings in low- and middle-income countries. Third, this study aims to provide a comprehensive overview of LLM applications in health care communication. To ensure the comprehensiveness of literature inclusion, we did not conduct a quality assessment of the included studies. Fourth, the rapidly evolving nature of LLMs means our findings primarily reflect the landscape as of the search date; subsequent new models and applications may alter current patterns.

### Conclusion

This review is the first to systematically summarize the application patterns of LLMs in health care communication by applying CAT. Currently, LLMs are still in the early stages of integration into clinical practice, and their widespread adoption continues to face challenges, including technical reliability, social trust and acceptance, barriers to interaction and access, and clinical integration. Future research should focus on optimizing model performance, strengthening ethical governance frameworks, and refining human-machine collaboration models, while ensuring safe application in the health care sector through rigorous empirical validation. This study highlights the potential of LLMs to optimize health care communication and is expected to promote their responsible application and high-quality development in medical practice.

## Supplementary material

10.2196/84726 Multimedia Appendix 1 Database search strategies, types of studies excluded, characteristics of included studies, and existing challenges.

10.2196/84726 Checklist 1 PRISMA-ScR checklist.

10.2196/84726 Checklist 2 PRISMA-S checklist.

## Abbreviations

AI: artificial intelligence
CAT: communication accommodation theory
LLM: large language model
MeSH: Medical Subject Headings
PRISMA: Preferred Reporting Items for Systematic Reviews and Meta-Analyses
PRISMA-S: Preferred Reporting Items for Systematic Reviews and Meta-Analyses literature search extension
PRISMA-ScR: Preferred Reporting Items for Systematic Reviews and Meta-Analyses extension for Scoping Reviews
