# Supplementary material for: Applications, Challenges, and Future Directions of Large Language Models in Health Care Communication: Scoping Review
Source: J Med Internet Res. 2026 Jun 26;28:e84726. doi: 10.2196/84726 (PMC13308756; doi:10.2196/84726)
Supplement: Multimedia Appendix 1 [file jmir-v28-e84726-s001.docx]

Contents list

1. Search strategies
2. Excluded category
3. Characteristics of included publications
4. Existing challenges and recommendations for the future

**1. Search strategies**

**Pubmed**

| S1 | **large language model* [Title/Abstract] OR language neural network* [Title/Abstract] OR chatgpt[MeSH Terms] OR language model* [Title/Abstract] OR language neural network* [Title/Abstract] OR generative ai [Title/Abstract] OR AI-Generated [Title/Abstract] OR generative artificial intelligence [Title/Abstract] chatgpt [Title/Abstract] OR Artificial Intelligence Chatbots [Title/Abstract] medpalm [Title/Abstract] OR gpt [Title/Abstract] OR pretrained model* [Title/Abstract] OR conversational ai [Title/Abstract] OR deep learning language model* [Title/Abstract] OR language generation model* [Title/Abstract] OR large language model[MeSH Terms]** |
| --- | --- |
| S2 | Respon* [Title] **OR** Repl* [Title] **OR** Report* [Title]**OR** Question* [Title] **OR** Transform* [Title] **OR** Summar* [Title] **OR** Communicat* [Title] **OR** Interpret* [Title] **OR** Explan* [Title] **OR** Inform* [Title] **OR** answer [Title] |
| S3 | S1 AND S2 Filters: **from 2018 - 2025** |

**Cochrane Library**

| S1 | (**large language model** OR **chatgpt**) explode all trees |
| --- | --- |
| S2 | (large language model* OR language neural network* OR generative ai OR AI-Generated OR generative artificial intelligence OR chatgpt OR Artificial Intelligence Chatbots OR medpalm OR gpt OR pretrained model* OR conversational ai OR deep learning language model* OR language model* OR language generation model*):ti,ab,kw |
| S3 | S1 OR S2 |
| S4 | (Respon* OR Repl* OR Report* OR Question* OR Transform* OR Summar* OR Communicat* OR Interpret* OR Explan* OR Inform* OR answer):ti,ab,kw |
| S5 | S3 AND S4 with Cochrane Library publication date from Jan 2018 to Nov 2025 |

**Web of science**

| 1 | TS=(**large language model** OR **chatgpt**) and Preprint Citation Index |
| --- | --- |
| 2 | TI=(large language model* OR language neural network* OR generative ai OR AI-Generated OR generative artificial intelligence OR chatgpt OR Artificial Intelligence Chatbots OR medpalm OR gpt OR pretrained model* OR conversational ai OR deep learning language model* OR language model* OR language generation model*) and Preprint Citation Index |
| 3 | S1 OR S2 |
| 4 | TI=(Respon* OR Repl* OR Report* OR Question* OR Transform* OR Summar* OR Communicat* OR Interpret* OR Explan* OR Inform* OR answer) |
| 5 | S3 AND S4 and Preprint Citation Index (Exclude – Database) and 2025 or 2024 or 2023 or 2022 or 2021 or 2020 or 2019 or 2018 (Publication Years) |

**Embase(OVID)**

| S1 | respon*:ti,ab,kw OR repl*:ti,ab,kw OR report*:ti,ab,kw OR question*:ti,ab,kw OR transform*:ti,ab,kw OR summar*:ti,ab,kw OR communicat*:ti,ab,kw OR interpret*:ti,ab,kw OR explan*:ti,ab,kw OR inform*:ti,ab,kw OR answer:ti,ab,kw |
| --- | --- |
| S2 | 'large language model'/exp OR ' chatgpt '/exp |
| S3 | 'large language model*':ab,kw,ti OR ' language neural network*':ab,kw,ti OR 'generative ai':ab,kw,ti OR 'AI-Generated':ab,kw,ti OR ' generative artificial intelligence ':ab,kw,ti OR ' chatgpt ':ab,kw,ti OR ' Artificial Intelligence Chatbots':ab,kw,ti OR 'medpalm':ab,kw,ti OR 'gpt':ab,kw,ti OR 'pretrained model*':ab,kw,ti OR ' conversational ai ':ab,kw,ti OR ' deep learning language model*':ab,kw,ti OR ' language model*':ab,kw,ti OR ' language generation model*':ab,kw,ti |
| S3 | S2 OR S3 |
| S4 | S1 AND S3 AND [2018-2025]/py |

# 2.Excluded category

| Excluded category | Typical content | Rationale for exclusion |
| --- | --- | --- |
| Medical knowledge test | Assessing LLM performance on standardized medical exams(e.g., USMLE). | These studies evaluate the internal knowledge repository rather than communication skills in clinical settings. |
| Clinical Data Extraction | The accuracy of using LLMs to extract features from medical texts, such as medical records | This falls under clinical informatics, not clinical communication. |
| Accuracy of Imaging Interpretation | Interpreting images and drawing conclusions | This constitutes diagnostic assistance and should not be included. |

Note: USMLE: United States Medical Licensing Examination

# 3.Characteristics of included publications

| **Author, year** | **Country location** | **Study design** | **Type of disease** | **Target population** | **Study objective** | **Type of the model** | **Evaluation Method** | **Outcomes Assessed** |
| --- | --- | --- | --- | --- | --- | --- | --- | --- |
| Pap et al. (2024) [67] | Romania | **Technical development study** | Generic | Patients | Develop an artificial intelligence health assistant | Based on ChatGPT | Inapplicable | Inapplicable |
| Warin et al. (2025) [121] | Thailand | Technical development study | Dentistry | Patients | To develop an AI chatbot for dental surgery information support | AI chatbot | Inapplicable | Accuracy |
| Chandler et al. (2025) [66] | USA | Descriptive study | Primary healthcare | Patients | Exploring ChatGPT's role in women's health self-education | ChatGPT3.5 | Assessment of experts | Content alignment |
| Bilgin et al. (2024) [69] | USA | Descriptive study | Oncology | Patients | Evaluate the ability of LLMs to answer prostate cancer questions | ChatGPT | Assessment of experts | Clarity, accuracy, readability, |
| Şan et al. (2024) [89] | Turkey | Descriptive study | Oncology | Patients | Evaluate the ability of LLMs to provide information about cancer | ChatGPT4, Google Bard | Assessment of tools | Reliability |
| Akçay et al. (2025) [68] | USA | Descriptive study | Oncology | Patients | Evaluate the ability of ChatGPT in answering patients' questions | ChatGPT | Assessment of experts | Accuracy clarity |
| Cicek et al. (2025) [70] | Turkey | Comparative study | Dentistry | Patients | Evaluate the response of LLMs to endodontic root canal treatment | DeepSeek V3, ChatGPT5, Gemini 2.5 | Assessment of experts | Accuracy, comprehensiveness |
| Dogan et al. (2025) [71] | Turkey | Descriptive study | Ophthalmology | Patients | Evaluate the ability of LLMs in frequently asked questions about glaucoma | ChatGPT4, Bing Chat | Assessment of tools | Readability, accuracy |
| Gencer et al. (2025) [72] | Turkey | Comparative study | Orthopedic | Patients | Evaluate the response of LLMs to endodontic root canal treatment sexual activity after total hip arthroplasty | ChatGPT | Assessment of experts | Clarity, consistency, accuracy |
| Goshtasbi et al. (2025) [73] | USA | Comparative study | Plastic Surgery | Patients | Comparative analysis of the response of LLMs in answering complex rhinoplasty consultation questions | ChatGPT4, Gemini, Claude, Meta-AI | Assessment of experts and tools | Readability |
| Gültekin et al. (2025) [74] | Turkey | Comparative study | Orthopedic | Patients | Evaluating LLMs in anterior cruciate ligament surgery patient education | ChatGPT, DeepSeek | Assessment of tools | Consistency, accuracy, clarity, comprehensiveness |
| Guven et al. (2025) [75] | Turkey | Comparative study | Dentistry | Patients | Evaluate the response of LLMs to traumatic dental injuries | ChatGPT3.5ChatGPT4,Gemini | Assessment of tools | Readability, quality, accuracy, understandability, actionability |
| He et al. (2025) [97] | China | Comparative study | Gastroenterology | Patients | Evaluate the response of LLMs to the recommendations related to inflammatory bowel disease | ChatGPT | Assessment of experts and patients | Comprehensiveness, readability, accuracy, personalization |
| Ibas et al. (2025) [76] | Turkey | Descriptive study | Plastic Surgery | Patients | Evaluate the response of LLMs to rhinoplasty postoperative counseling | ChatGPT | Assessment of experts | Accuracy, safety |
| Kamminga et al. (2025) [77] | Netherlands | Comparative study | Dermatology | Patients | Evaluate the response of LLMs in answering patient questions | ChatGPT3.5, ChatGPT4, Gemini | Assessment of experts | Comprehensiveness, accuracy, personalization, readability |
| Lewandowski et al. (2025) [79] | Poland | Comparative study | Dermatology | Patients | Evaluate the response of LLMs to hidradenitis suppurativa | ChatGPT4 | Assessment of experts and patients | Quality, empathy, satisfaction |
| Li et al. (2025) [80] | USA | Descriptive study | Orthopedic | Patients | Evaluate the response of LLMs to pediatric supracondylar humerus fractures | ChatGPT | Assessment of experts | Comprehensiveness, accuracy |
| Guo et al. (2025) [81] | China | Comparative study | Surgery | Patients | Evaluate the response of LLMs to thyroid questions | ChatGPT4, ChatGPT4omni | Assessment of experts and patients | Response speed, accuracy, comprehensiveness, empathy, satisfaction |
| Mete et al. (2025) [82] | Turkey | Descriptive study | Otolaryngology | Patients | Evaluate the response of LLMs toinquiries about otosclerosis | ChatGPT | Assessment of experts | Accuracy |
| Moothedan et al. (2025) [83] | USA | Descriptive study | Infectious Diseases | Patients | Evaluate the response of LLMs to sexually transmitted diseases | ChatGPT | Assessment of experts | Accuracy, clarity, appropriateness |
| Motegi et al. (2025) [84] | Japan | Comparative study | Ophthalmology | Patients | Comparison of the response of LLMs to online ear, nose, and throat inquiries | ChatGPT4 | Assessment of experts | Quality, empathy, consistency, accuracy, comprehensiveness, harm potential |
| Özcivelek et al. (2025) [86] | Turkey | Comparative study | Dentistry | Patients | Evaluate the response of LLMs to dental and maxillofacial prostheses | DeepSeek, ChatGPT, dental GPT chatbots | Assessment of experts and tools | Accuracy, quality, understandability, actionability |
| Raghunathan et al. (2025) [86] | USA | Cross-sectional study | Surgery | Patients | Evaluate the response of LLMs to unmet patient information needs in the management of thyroid disease | ChatGPT3.5, ChatGPT4 | Assessment of experts | Accuracy, quality, empathy |
| Rotem et al. (2025) [87] | Ireland | Comparative study | Urogynecology | Patients | Comparison of LLM and human experts answering urogynecological questions | ChatGPT | Assessment of experts and patients | Understandability, helpfulness, reassurance |
| Roy et al. (2025) [88] | USA | Comparative study | Surgery | Patients | Comparison of LLM and human experts answering cerebrovascular neurosurgical questions | ChatGPT | Assessment of experts | Quality, empathy, readability |
| Scaff et al. (2025) [90] | Brazil | Cross-sectional study | Orthopedic | Patients | Evaluate the response of LLMs to low back pain | ChatGPT3, ChatGPT4, Bing, Gemini | Assessment of experts | Accuracy, readability, |
| Shao et al. (2025) [91] | China | Comparative study | Surgery | Patients | Evaluate the response of LLMs to gynecomastia inquiries | ChatGPT, DeepSeek, Gemini, Perplexity, Copilot | Assessment of tools | Quality, readability, |
| Tuzlalı et al. (2025) [92] | Turkey | Comparative study | Dentistry | Patients | Evaluate the response of LLMs to dental implant FAQs | ChatGPT, DeepsSeek, Gemini, Claude, Perplexity | Assessment of experts and layman | Accuracy, comprehensiveness, clarity, relevance, consistency |
| Weber et al. (2025) [93] | Germany | Comparative study | Respiratory Medicine | Patients | Assess the efficacy of LLMs in physician-patient communication for rare diseases | ChatGPT4; BioMistral 7B | Assessment of experts | Correctness, comprehensibility, relevance, empathy |
| White et al. (2025) [94] | USA | Descriptive study | Orthopedic | Patients | Assess the response of LLMs to total shoulder arthroplasty | ChatGPT | Assessment of experts | Quality, accuracy |
| Wu et al. (2025) [98] | China | Comparative study | Respiratory Medicine | Patients | LLM's responses to influenza patient questions | Not mentioned | Assessment of experts | Extent of harm, empathy |
| Xue et al. (2025) [95] | China | Comparative study | Obstetrics | Patients | Comparing the response of LLMs to post-abortion care | ChatGPT, Kimi, Ernie Bot | Assessment of experts | Accuracy, relevance, Comprehensiveness, clarity, reliability |
| Zhang et al. (2025) [96] | Singapore | Descriptive study | Orthopedic | Patients | Assess personalized responses of LLMs to anterior cruciate ligament reconstruction | ChatGPT | Assessment of experts | Accuracy, the degree to which it was personalized |
| Gondode et al. (2024) [61] | India | Comparative study | Generic | Patients | Evaluate the ability of LLMS to generate information handbooks for patients in end-of-life care | ChatGPT Google Gemini | Assessment of experts and tools | Readability, emotion, accuracy, comprehensiveness, applicability |
| Kianian et al. (2024) [62] | USA | Descriptive study | Urology | Patients | Evaluate the ability of LLMS to generate health information for patients | ChatGPT | Assessment of tools | Quality, readability |
| Vallurupalli et al. (2024) [59] | USA | Descriptive study | Surgery | Patients | Evaluate the ability of LLMs to optimize educational materials for patients' hand surgery | ChatGPT3.5 | Assessment of tools | Readability |
| Chandra et al. (2025) [60] | USA | Descriptive study | Surgery | Patients | Evaluate the ability of LLMs to optimize educational materials for patients' shoulder and elbow surgeries | ChatGPT4 | Assessment of tools | Readability, accuracy |
| Chung et al. (2023) [41] | USA | Descriptive study | Radiology | Patients | Evaluating LLMs for generating radiology report summaries for cancer patients | ChatGPT | Assessment of experts and tools | Understanding, Comprehensiveness, the potential for harm, overall quality, likelihood to send to patient |
| Li et al. (2023) [46] | USA | Descriptive study | Radiology | Patients | Evaluating LLMs for simplifying radiology report summaries | ChatGPT | Assessment of tools | Readability |
| Sarangi et al. (2023) [49] | India | Descriptive study | Radiology | Clinicians &patients | Evaluating LLMs in simplifying radiological reports for healthcare professionals and patients | ChatGPT 3.5 | Assessment of experts | Comprehensiveness, accuracy, quality |
| Butler et al. (2024) [39] | USA | Descriptive study | Radiology | Patients | Improving the readability of foot and ankle radiology reports with LLMs | AI-LLM | Assessment of tools | Readability |
| Gulati et al. (2024) [65] | USA | Descriptive study | Radiology | Patients | To explore the capabilities of LLMs for the purpose of simplifying and translating | ChatGPT4 | Assessment of tools | Correctness, potentially, translation quality, comprehensiveness, |
| Kuckelman et al. (2024) [45] | USA | Descriptive study | Radiology | Patients | Evaluating LLMs to generate concise and accurate layperson summaries of musculoskeletal radiology reports | ChatGPT4 | Assessment of experts | Comprehensiveness; accuracy |
| Schmidt et al. (2024) [50] | Germany | Cohort study | Radiology | Patients | Evaluating LLMs in simplifying radiologic reports with LLMs | ChatGPT | Assessment of experts and laymen | Accuracy, comprehensiveness |
| Steimetz et al. (2024) [51] | USA | Cross-sectional study | Pathology | Patients | Use of LLMs in the interpretation of pathology reports | Bard, ChatGPT-4 | Assessment of experts | Accuracy, readability |
| Tang et al. (2024) [54] | USA | Cross-sectional study | Radiology | Patients | Use of LLMs in generating colloquial radiology reports | Not mentioned | Assessment of experts | Accuracy, likability, harm potential, readability |
| Zaretsky et al. (2024) [58] | USA | Descriptive study | Primary healthcare | Patients | LLMs to transform inpatient discharge summaries to patient-friendly language and format | ChatGPT4 | Assessment of experts | Understandability, readability, accuracy, comprehensiveness |
| Anand et al. (2025) [36] | UK | Pilot study | Radiology | Patients | LLMs to generate  patient-friendly Magnetic Resonance Imaging fistula summaries | ChatGPT4 | Assessment of experts | Readability, utility, quality, comprehensibility, hallucination |
| Berzolla et al. (2025) [39] | USA | Randomized controlled trial | Radiology | Patients | LLMs to improve patient comprehension of radiologists' magnetic resonance imaging reports | ChatGPT4 | Assessment of patients | Clarity, comprehension |
| Bheemireddy, et al. (2025) [38] | USA | Descriptive study | Pathology | Patients | LLMs to simplify breast pathology reports | ChatGPT4 | Assessment of experts | Readability, accuracy |
| Chen et al. (2025) [40] | USA | Randomized controlled trial | Radiology | Patients | Improving patient understanding of radiology reports using LLMs | ChatGPT4 | Assessment of experts and patients | Readability, accuracy |
| Dias et al. (2025) [64] | USA | Descriptive study | Oncology | Patients | Does ChatGPT 3.5 provide appropriate responses to frequently asked patient questions | ChatGPT3.5 | Assessment of experts | Comprehensibility |
| Eisinger et al. (2025) [42] | Germany | Exploratory study | Primary health care | Patients | Evaluate the potential of LLMs to transform discharge letters into patient-centered letters | ChatGPT-4 | Assessment of experts | Accuracy, the ability to identify and translate the learning objectives |
| Hains et al. (2025) [43] | Australia | Comparative study | Primary health care | Patients | Testing the efficacy of two LLMs to generate discharge letter summaries | llama3:ininstruction, llama3:70b | Assessment of tools | Quality |
| Juan-Guardela et al. (2025) [63] | Colombia | Cross-sectional study | Internal medicine | Patients | Evaluate the information provided by LLMs in English and Spanish for atrial fibrillation patients | ChatGPT, YouChat, Gemini; Perplexity | Assessment of experts | Reliability, readability |
| Khanmammadova et al. (2025) [44] | USA | Quality Improvement study | Oncology | Patients | Evaluation of prostate cancer pathology reports generated by LLMs | ChatGPT3.5 | Assessment of experts and patients | Readability |
| Li et al. (2025) [47] | USA | Comparative study | Primary health care | Patients | Evaluation of hospital discharge summaries for lung cancer patients by LLMs | ChatGPT3.5ChatGPT4ChatGPT4o, LLaMA 3 8b | Assessment of experts | Clinical relevance, Comprehensiveness |
| Prucker et al. (2025) [48] | Germany | Comparative study | Radiology | Patients | Compare LLMs in generating patient-friendly radiology chest CT reports | Llama3-70b, Mistral-7b, Mixtral8x7b ChatGPT4, GPT3.5, Claude3, Gemini | Assessment of experts | Reliability, accuracy, readability |
| Stephan et al. (2025) [52] | Germany | Comparative study | Radiology | Patients | Simplifying AI-Generated dental radiology reports with LLMs | ChatGPT | Assessment of patients | Readability |
| Sunshine et al. (2025) [53] | USA | Survey study | Radiology | Patients | Evaluating the performance of radiology report summaries generated by LLMs | ChatGPT | Assessment of experts and patients | Quality, accuracy, comprehensibility, satisfaction, readability |
| Williams et al. (2025) [55] | USA | Cross-sectional study | Primary healthcare | Patients | Evaluating the performance of hospital discharge summaries generated by LLMs | ChatGPT4 | Assessment of experts | Quality, reviewer preference, comprehensiveness, hallucinations |
| Yang et al. (2025) [56] | China | Descriptive study | Pathology | Patients | Using LLMs for pathology report interpretation | ChatGPT4 | Assessment of patients | Consistency, readability |
| Yang et al. (2025) [57] | China | Multicenter quantitative Study | Oncology | Patients | Using LLMs Simplified Radiology Reports | ChatGPT4 | Assessment of expert patients | Consistency, readability, communication efficiency |
| Cai et al (2024) [113] | USA | Technical development study | Neurology | Patients | Using LLMs to accelerate communication for eye gaze typing users with amyotrophic lateral sclerosis | LaMDA | Inapplicable | Typing speed, text-entry rates |
| Adikari et al (2025) [112] | Australia | Technical development study | Neurology | Patients | Reconstructing impaired language using LLMs for people with aphasia | ChatGPT4 | Inapplicable | Accuracy |
| Muasher-Kerwin et al (2025) [99] | USA | Descriptive study | Surgery | Patients | Exploring the application of large language models in summarizing and explaining cancer-related information to bridge communication gaps | ChatGPT, Llama 3 | Assessment of tools | Readability, accuracy |
| Kienzle et al. (2024) [103] | Germany | Descriptive study | Orthopedics | Clinicians &patients | To test the performance of informed consent of patients before LLMs replacement for total knee arthroplasty | ChatGPT | Assessment of tools | Clarity, reliability, overall quality |
| Allen et al. (2026) [101] | Australia | Proof of concept Study | Generic | Patients | Testing the role of LLMs in scaffolding Informed Consent in Medical Practice | Not mentioned | Inapplicable | Inapplicable |
| Bertges et al. (2025) [100] | USA | Descriptive study | Oncology | Clinicians &patients | Testing LLM's ability to provide patient and physician information on aortic aneurysm | ChatGPT4 | Assessment of experts | Accuracy, quality |
| Grünebaum et al. (2025) [102] | USA | Descriptive study | Obstetrics | Patients | Explore the role of LLMs in simplifying informed consent for labor induction with oxytocin | ChatGPT4, Claude | Assessment of tools | Accuracy, readability |
| Chung et al. (2024) [116] | USA | Descriptive study | Surgery | Clinicians &patients | Testing LLM's ability to provide in Pre-Vasectomy counseling | ChatGPT4 | Assessment of experts | Quality, accuracy |
| Kuo et al. (2024) [78] | USA | Cross-sectional study | Anesthesia | Patients | Comparing LLMs and anesthesiologists' responses to common patient questions | ChatGPT3.5 | Assessment of experts | Quality, emotion |
| Wan et al. (2024) [114] | China | Randomized controlled trail | Generic | Nurses | Outpatient reception via collaboration between nurses and an LLM | SPEEC | Assessment of experts and laymen | Factuality, integrity, readability, empathy, safety, satisfaction |
| Carl et al. (2025) [115] | Germany | Descriptive study | Surgery | Patients | Investigating medical information provided by LLMs | ChatGPT4 | Assessment of patients | Utility, comprehensiveness, understandability |
| Park et al. (2025) [117] | Korea | Comparative study | Emergency | Patients | To explore the clinical performance and communication skills of LLMs in emergency medicine | ChatGPT | Assessment of experts | History taking, accuracy, empathy |
| Santonocito et al. (2025) [118] | Italy | Randomized controlled trial | Dentistry | Patients | Evaluation of the LLMs on orthodontic patient education | AI chatbot | Assessment of patients | Knowledge, satisfaction |
| Scholich et al. (2025) [119] | USA | Mixed-methods study | Psychology | Patients | Comparison of responses of human therapists and LLMs in assessing therapeutic communication | ChatGPT, Pi, Replika | Qualitative interview | Inapplicable |
| Gutiérrez et al. (2024) [107] | Australia | Usability study | Emergency | Clinicians | Using LLMs to facilitate communication training among medical emergency personnel | ChatGPT | Assessment of tools | User experience, usability |
| Chen (2025) [105] | Taiwan, China | Randomized controlled trial | Obstetrics | Nursing students | Explore the effect of combining LLMs with virtual reality for maternal communication simulation | ChatGPT | Assessment of tools | Empathy, communication confidence, communication skills |
| Chen et al. (2025) [106] | Taiwan, China | Mixed-methods study | Obstetrics | Nursing students | Application of LLMs-driven interactive virtual reality communication simulation in obstetric care | ChatGPT | Assessment of tools | Communication confidence, communication skills, system availability |
| Kim et al. (2025) [108] | Korea | Mixed-methods study | Generic | Nursing students | Use of a LLMs-based virtual patient for health assessment and communication training in nursing education | ChatGPT | Qualitative interview | Usability, communication self-Efficacy |
| Wang et al. (2025) [109] | China | Feasibility study | Generic | Medical Students | Using LLMs for history-taking training in medical education | ChatGPT | Structured clinical examination | Educational effectiveness, satisfaction, the likelihood of recommendation |
| Weisman et al. (2025) [110] | USA | Multiphase study | Gynecology | Medical Students | Development of an LLMs-Powered virtual simulated patient and communication training platform | ChatGPT4 | Qualitative interview | Quality of responses |
| Bakhaya et al. (2026) [104] | Sweden | Qualitative study | Pharmacy | Nursing students | Development and evaluation of an LLM tool for self-care written communication training | ChatGPT | Qualitative interview | Perceived usefulness |
| Genes et al. (2025) [122] | USA | Descriptive study | Emergency | Clinicians | LLMs summaries to facilitate emergency department handoff | ChatGPT4 | Assessment of experts | Accuracy, clinical efficacy, safety |
| Chen et al. (2025) [121] | China | Technical development study | Emergency | Clinicians | Generation of prehospital emergency diagnostic summaries using LLMs | Qwen2.5-7B-Ininstruction | Not mentioned | Quality |
| Tailor et al. (2025) [123] | USA | Quality improvement study | Ophthalmology | Clinicians | Evaluating the effectiveness of LLM-Generated summaries integrated into standardized ophthalmic notes (SONs) | Not mentioned | Assessment of experts | Understanding, satisfaction, clarity, quality, safety analysis, clinical workflow, accuracy |
| Chen et al. (2025) [111] | China | Technical development study | Generic | Clinicians | LLMs were used to assist medical record generation and optimize emotional EEG interpretation | Not mentioned | Inapplicable | Inapplicable |
| English et al. (2024) [130] | USA | Quality improvement study | Generic | Clinicians | Generate draft responses for patients using LLMs | ChatGPT4 | Assessment of experts | Attitudes toward LLMs |
| Garcia et al. (2024) [124] | USA | Quality improvement study | Generic | Clinicians | Generate draft responses for patients using LLMs | ChatGPT4 | Assessment of experts | A percentage of total patient message replies, changes in time measures and clinician experience |
| Liu et al. (2024) [126] | USA | Descriptive study | Generic | Clinicians | Generate draft responses for patients using LLMs | CLAIR-Short, CLAIR-long ChatGPT | Assessment of experts | Empathy, responsiveness, accuracy, usefulness. |
| Scott, et al. (2024) [127] | USA | Descriptive study | Urology | Clinicians | Generate draft responses for patients using LLMs | ChatGPT | Assessment of experts | Accuracy, Comprehensiveness, helpfulness, |
| Small et al. (2024) [128] | USA | Quality improvement study | Generic | Clinicians | Generate draft responses for patients using LLMs | ChatGPT | Assessment of experts | Content quality, communication quality, empathy, personalization, |
| Soroudi et al. (2024) [129] | USA | Quality improvement study | Surgery | Clinicians | Generate draft responses for patients using LLMs | ChatGPT3, ChatGPT4 | Assessment of experts | Accuracy, empathy, respectively, readability |
| Kaur et al. (2025) [125] | USA | Cross-sectional study | Generic | Clinicians | Automating responses to patient portal messages with LLMs | ChatGPT3.5 | Assessment of experts and patients | Accuracy, empathy, readability |
| Mandal et al. (2025)[131] | USA | Observational study | Generic | Clinicians | Generate draft responses for patients using LLMs | ChatGPT3, ChatGPT4 | Inapplicable | GenAI draft utilization |

# 4.Existing challenges and recommendations for the future

| **Existing challenges** |  | **Recommendation for the future** |
| --- | --- | --- |
| **Technical**  **reliability** | Hallucinations & omission | - Build dynamic datasets (knowledge graphs & multimodal data) |
|  | Insufficient transparency and timeliness | - Integrated retrieval-enhanced generation frameworks |
| **Social trust and adoption** | Emotional constraints & Lack of depth | - Develop multimodal systems - Optimize human-AI collaboration |
|  | Fairness dilemma | - Build diverse, unbiased datasets |
|  | legal-ethical concerns | - Introducing differential privacy technology - Establish a comprehensive legal framework |
| **Interaction and access barriers** | Quality of user prompts varies | - Building a prompt optimization support framework |
|  | Resource inequality | - Develop public education courses |
| **Clinical integration challenges** | Insufficient standardization and personalization | - Develop standardized and structured templates - Patient data secure access |
|  | Security Risk | - Establish risk identification and response mechanisms - Artificial intervention channel |
